# Effect of seed-applied fluopyram on *Meloidogyne incognita* infection and maturity in cotton and soybean

**DOI:** 10.21307/jofnem-2020-083

**Published:** 2020-08-25

**Authors:** Tracy Hawk, Travis R. Faske

**Affiliations:** Division of Agriculture, Lonoke Extension Center, University of Arkansas, Lonoke, AR, 72086

**Keywords:** Abamectin, Behavior, Management, Seed treatment

## Abstract

Fluopyram is being used to manage plant-parasitic nematodes in cotton (*Gossypium hirsutum*) and soybean (*Glycine max*), but the duration and depth of root protection from *Meloidogyne incognita* by seed-applied fluopyram is unknown. Both *M. incognita* susceptible cotton, Stoneville ‘ST 4848 GLT’, and soybean, Delta Grow ‘DG 4880 GLY’, cultivars were treated with fluopyram or abamectin and inoculated with second-stage juveniles in two greenhouse studies. Root penetration by *M. incognita* was suppressed from 7 to 21 d after planting by seed-applied fluopyram in soybean, while a similar trend in suppression was observed in cotton. Fewer nematodes per root system by fluopyram contributed to a reduction in root gall counts and nematode reproduction at 28 and 35 d after planting in both crops. Based on nematode developmental stages from 7 to 21 d after planting, fluopyram had no effect on nematode maturity. Root penetration by *M. incognita* was suppressed at 7 d after planting by fluopyram at a depth up to 5.0 cm in cotton and 2.5 cm in soybean. These results were similar to that of abamectin-treated seed. Seed-applied fluopyram and abamectin were most effective at suppressing nematode root entry rather than nematode maturity in cotton and soybean.

The southern root-knot nematode, *Meloidogyne incognita* (Kofoid and White) Chitwood race 3, is an important yield-limiting pest of cotton (*Gossypium hirsutum* L.) and soybean (*Glycine max* (L.) Merr.) in the southern USA (Thomas and Kirkpatrick, 2001; Koenning, 2015). Furthermore, it is the most prevalent species of root-knot nematode in Arkansas (Ye et al., 2019). Seed cotton losses in 2018 were estimated at 2.4% (966,600 bales) across the US Cotton Belt (Lawrence et al., 2019). While non-soybean cyst nematodes (including *Meloidogyne* spp.) were the second most destructive pathogen on soybean in 2014 in the southern USA, with an estimated grain yield loss of 18 million bushels (Allen et al., 2017).

Root-knot nematode management includes a combination of resistant cultivars, crop rotation, and nematicides. Currently, a few soybean and cotton cultivars are available with a moderate magnitude of resistance to *M. incognita* (Emerson et al., 2018; Wheeler et al., 2018). However, because of limited availability to a specific herbicide technology, maturity group, or lack of a competitive yield potential these *M. incognita*-resistant cultivars are often underutilized. Crop rotation with a non-host crop is an effective tool to manage *M. incognita* population densities. Peanut (*Arachis hypogaea* L.) is the only non-host crop grown in the Mid-South and is currently produced on a limited number of acres compared to that of cotton and soybean (USDA-NASS, 2018).

During the past 15 years, the availability and use of seed-applied nematicides have increased in row crop agriculture. One of the more recent nematicides registered (2014) for use in cotton and soybean is fluopyram (Environmental Protection Agency (EPA), 2014). Fluopyram is a succinate dehydrogenase inhibitor fungicide that has been reported to affect the motility of *M. incognita* and other plant-parasitic nematodes (Faske and Hurd, 2015; Heiken, 2017; Beeman and Tylka, 2018). However, the field efficacy of fluopyram has been variable in *M. incognita* suppression and protecting cotton lint and soybean grain yield potential (Lawrence et al., 2016; Hurd et al., 2017; Jackson, et al., 2017; Faske et al., 2018). Further characterization of root protection by seed-applied fluopyram is needed to understand the variability in nematode suppression.

The objectives of this study were to evaluate the effect of seed-applied fluopyram on *M. incognita* penetration, infection, and maturity of young cotton and soybean roots, and determine the depth of cotton and soybean root protection provided by fluopyram-treated seed.

## Materials and methods

### Nematode inoculum

Cultures of *Meloidogyne incognita* race 3, collected from cotton (Leachville, AR) and soybean (Kerr, AR) were maintained on tomato (*Solanum lycopersicum* L., ‘Rutgers’) in a greenhouse, where ambient temperatures ranged from 26 to 33°C. Eggs were extracted from tomato roots with 0.5% NaOCl (Hussey and Barker, 1973). Eggs were placed in a hatching chamber (Vrain, 1977) in an incubator set to 28°C and second-stage juveniles (J2) were collected every 24 hr. Only 24-48-hr-old J2 were used as inoculum.

### Cotton and soybean treated seed

A southern root-knot nematode susceptible cotton cultivar, Stoneville ‘ST 4848 GLT’ (BASF Corporation, Florham Park, NJ) was used in this study. All cotton seed treatments were applied by the manufacturer and treated with a base fungicide of 15 μg metalaxyl/seed + 5 μg penflufen/seed + 5 μg prothioconazole/seed + 3 μg myclobutanil/seed [Allegiance^®^ FL + EverGol^®^ Prime + Proline^®^ 480 SC (Bayer CropScience, Research Triangle Park, NC) + Spera™ 240 FS (Nufarm Americas Inc., Alsip, IL), respectively]. Nematicide treatments included fluopyram at 200 μg/seed (Copeo^®^ Prime 600 FS, BASF Corporation) and abamectin at 150 μg/seed (Avicta^®^ 500 FS, Syngenta Crop Protection, Greensboro, NC) + 340 μg thiamethoxam/seed (Cruiser^®^ 5 FS, Syngenta Crop Protection). Fungicide treated seed was used as the non-treated control.

A southern root-knot nematode susceptible soybean cultivar, Delta Grow ‘DG 4880 GLY’ (Delta Grow Seed Co. Inc., England, AR) was used in this study (Emerson et al., 2018). Pesticides were applied with a rotary seed treating system (UNICOAT 1200 CCS, Universal Coating Systems, Inc., Independence, OR). Fluopyram-treated seed included 150 μg fluopyram/seed (ILEVO^®^ 600 FS, BASF Corporation) + 120 μg imidacloprid/seed (Gaucho^®^ 600 FS, Bayer CropScience). Abamectin-treated seed received 150 μg abamectin/seed (Avicta^®^ 500 FS, Syngenta Crop Protection) + 150 μg thiamethoxam/seed (Cruiser^®^ 5 FS, Syngenta Crop Protection). The non-treated control seed consisted of 120 μg imidacloprid/seed (Gaucho^®^ 600 FS, Bayer CropScience).

### Time course experiments

A time course experiment was conducted to evaluate the effect of seed-applied fluopyram on suppression of *M. incognita* infection, development, and reproduction in a greenhouse. Two seed treatments, fluopyram and abamectin, were compared in the time course experiments on cotton and soybean. The time course series consisted of roots sampled at 7, 14, 21, 28, and 35 DAP. All seeds were planted in a course (< 2.0-mm-diameter) pasteurized sand (90% sand, 6% silt, 4% clay; pH 7.3; cation exchange capacity 3.6 cmol+/kg). Sand was blended with inoculum at a rate of 2 J2/cm^3^ soil in 164 cm^3^ and 656 cm^3^ Deepots (Stuewe and Sons, Inc, Corvallis, OR). One seed of cotton or soybean was planted at a depth of 1.75-cm per pot. To promote uniform germination and minimize water evaporation, pots were covered with a transparent plastic wrap for 3 d at 30°C until seedling emergence. Seedlings were moved onto a greenhouse bench where ambient temperatures ranged from 26 to 33°C. Plants were overhead watered daily and fertilized every 10 d with 1.85 cm^3^ of Osmocote 20-20-20 (Scotts Miracle-Gro, Marysville, OH). Treatments were arranged in a randomized complete block design with five replications per treatment per sample time. The experiment was conducted twice for cotton and soybean.

**Figure 1:**
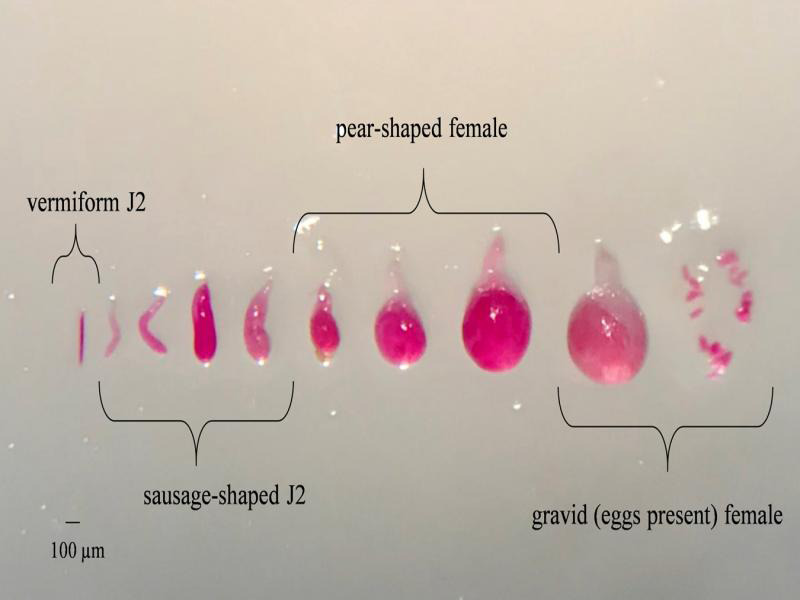
Various life stages of *Meloidogyne incognita* stained with acid fuchsin. Life stages include vermiform second-stage juvenille (J2), sausage-shaped juvenile, pear-shaped female, and gravid (eggs present) female.

Roots sampled at 7, 14, and 21 d after planting (DAP) from small Deepots were stained with acid fuchsin (Byrd et al., 1983). Stained nematodes were enumerated using a stereomicroscope and categorized into four different maturity stages: vermiform J2, sausage-shaped juvenile, pyriform (pear-shaped) female, and gravid (eggs present) female (Fig. 1) (Karssen and Moens, 2006). Of the developmental stages assessed, the majority fell into two categories per sample time, thus only two stages are reported as a percentage of nematode maturity stages per root system. Roots sampled at 21, 28, and 35 DAP from large Deepots were assessed for number of galls and roots from 28 and 35 DAP were assessed for number of eggs per g or root. Eggs were extracted with 1% NaOCl and enumerated using a stereomicroscope. Experiments for both cotton and soybean were conducted twice.

### Depth of root protection experiments

To determine the depth of root protection provided by fluopyram-treated cotton and soybean seed, a laboratory assay was conducted. For this assay, 50-ml conical tubes (Thermo Fisher Scientific Inc., Waltham, MA) were filled with the sandy soil described above to a bulk density of 1.4 g/cm^3^. Soybean and cotton seeds were planted 2-cm deep and each tube received 5 ml of water. Seedlings were incubated at 26 to 33°C under a greenhouse bench and watered daily, as needed. Cotton and soybean roots were inoculated with 250 J2 in 200 μl of water at five DAP through a 1.5 mm-diam. hole bored into the side of tubes at a depth of 2.5, 5.0, or 7.0 cm. The treatments were arranged in a randomized complete block design with each treatment and inoculation depth replicated four times. The experiment was conducted twice per crop. In total, 48 hr after inoculation, roots were removed, rinsed with water, and stained with acid fuchsin (Byrd et al., 1983), and vermiform nematodes were enumerated using a stereomicroscope.

### Statistical analysis

Data were analyzed using general linear mixed model analysis of variance (ANOVA) with experiment and treatment as fixed variables and replication modeled as a random variable using IBM SPSS Statistics 25.0 (International Business Machines Crop., Armonk, NY). Data were log transformed [log(*x* + 1)] to normalize for analysis and non-transformed data are reported. Mean separation were based on Tukey’s honest significant difference (HSD) test at *α* = 0.05. Stages of nematode maturity data were subject to chi-square analysis using IBM SPSS.

## Results

**Figure 2:**
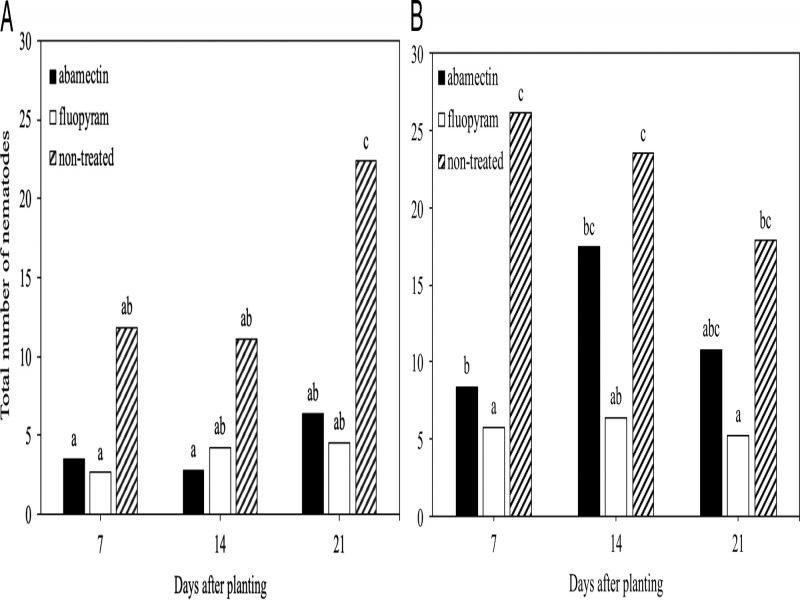
Suppression of *Meloidogyne incognita* at three sample times by seed-applied nematicides in cotton (A) and soybean (B). Different letters above bars indicate significant differences at *α* = 0.05 according to Tukey’s HSD test.

**Figure 3:**
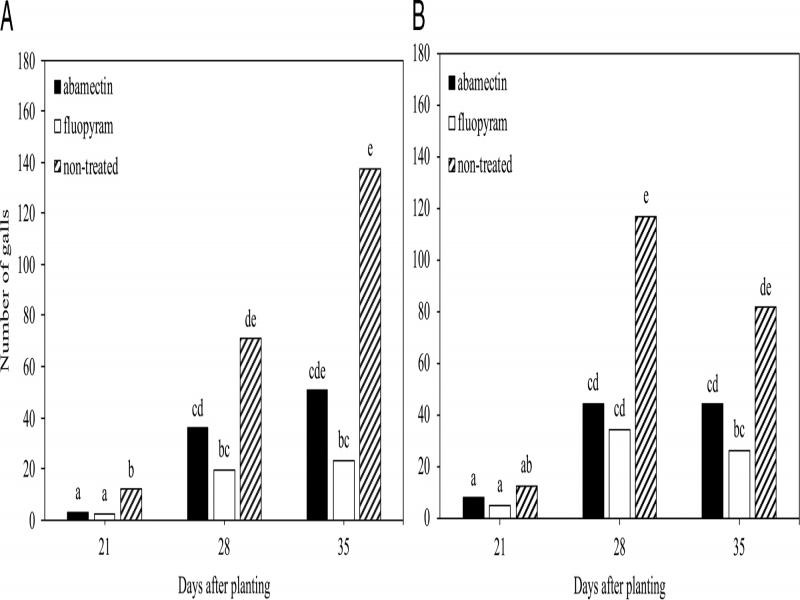
Suppression of *Meloidogyne incognita* root galling by seed-applied nematicides at three sample times on cotton (A) and soybean (B). Different letters above bars indicate significant differences at *α* = 0.05 according to Tukey’s HSD test.

**Figure 4:**
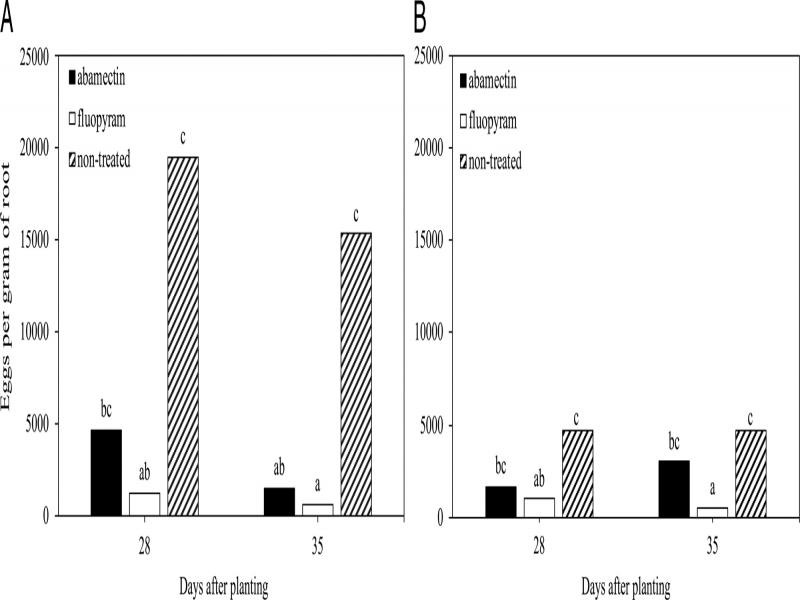
Reproduction of *Meloidogyne incognita* on cotton (A) and soybean (B) in response to seed-applied nematicides. Different letters above bars indicate significant differences at *α* = 0.05 according to Tukey’s HSD test.

In the time course experiments for cotton or soybean there was no (*P* > 0.05) experiment by treatment interaction for nematode root penetration, infection, or reproduction, so experiment repetitions per crop were combined for final analysis. When all developmental stages were combined, fewer (*P* ≤ 0.05) total nematodes were observed on cotton roots at 21 DAP with seed-applied abamectin and fluopyram compared to the non-treated control (Fig. 2A). While fewer (*P* ≤ 0.05) total nematodes were observed on soybean roots at 7, 14, and 21 DAP for fluopyram compared to the non-treated control (Fig. 2B). Fewer (*P* ≤ 0.05) galls were observed on cotton roots at 21, 28, and 35 DAP for fluopyram than the non-treated control, while inconsistent galling suppression was observed for abamectin (Fig. 3A). In soybean, no differences occurred between treatment groups at 21 DAP (Fig. 3B). Fewer (*P* ≤ 0.05) galls were observed at 28 and 35 DAP for fluopyram compared to the non-treated control, while inconsistent galling suppression was observed with abamectin (Fig. 3B). Nematode reproduction was lower (*P* ≤ 0.05) at 28 and 35 DAP for fluopyram and 35 DAP for abamectin compared to the non-treated control on cotton (Fig. 4A). In soybean, nematode reproduction was lower (*P* ≤ 0.05) at 28 and 35 DAP for fluopyram, but not for abamectin compared to the non-treated control (Fig. 4B).

**Figure 5:**
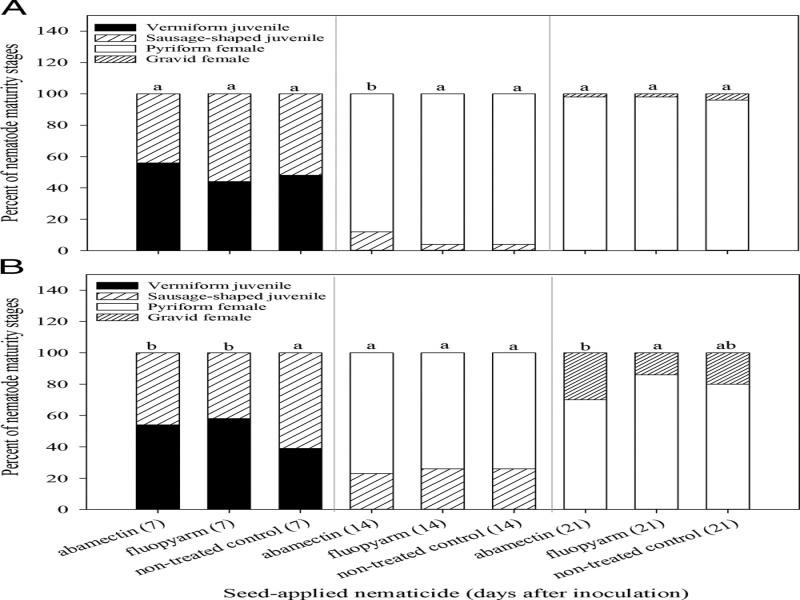
Post-infection maturity of *Meloidogyne incognita* on cotton (A) and soybean (B) in response to seed-applied nematicides. Different letters above bars indicate significant differences at *α* = 0.05 according to chi-square analysis applied in pairs of treatments within each sample time.

The percent of sausage-shaped juveniles and pyriform females at 14 DAP with abamectin differed (*P* ≤ 0.05) from that of fluopyram and the non-treated control on cotton, with more juveniles and less females in abamectin samples (Fig. 5A). However, that was the only time that nematode maturity differed in cotton. In soybean, the percent of vermiform and sausage-shaped juveniles differed (*P* ≤ 0.05) between abamectin and fluopyram compared to the non-treated control, with more vermiform nematodes present, however, that trend was inconsistent across sample times (Fig. 5B). Overall, neither abamectin nor fluopyram consistently affected the maturity of *M. incognita* in cotton or soybean. Thus, once *M. incognita* establishes a feeding site these nematicides have little impact on nematode maturity.

**Figure 6:**
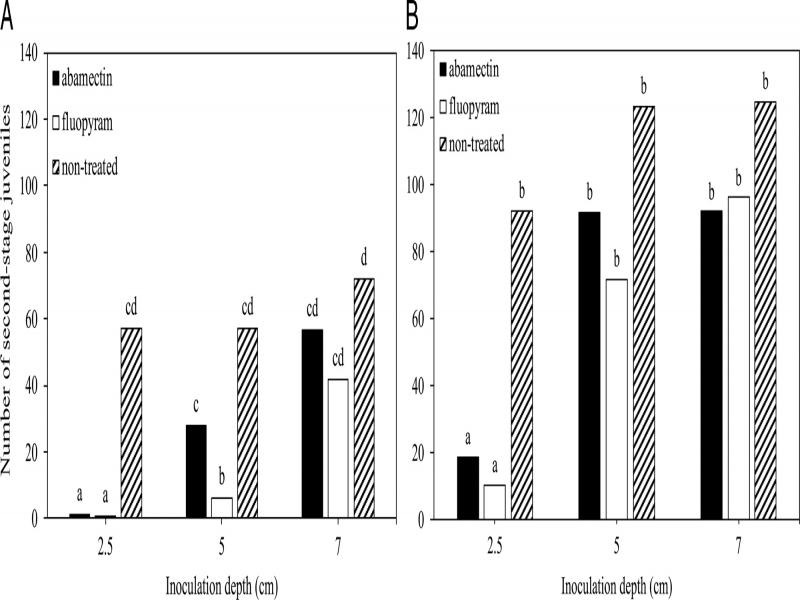
Suppression of *Meloidogyne incognita* root penetration at three inoculation depths by seed-applied nematicides in cotton (A) and soybean (B). Different letters above bars indicate significant differences according to Tukey’s HSD test at *α* = 0.05.

In the depth of root protection experiments, there was no experiment by treatment by inoculation depth interaction for cotton or soybean, however, there was an interaction (*P* ≤ 0.05) between treatment and inoculation depth for each crop. Fewer (*P* ≤ 0.05) J2 were observed in roots at 2.5 and 5.0 cm depth for fluopyram and only at 2.5 cm depth for abamectin compared to the non-treated control in cotton (Fig. 6A), whereas a similar number of J2 were observed at 7.0 cm depth for all treatments in cotton. In soybean, fewer (*P* ≤ 0.05) J2 were observed at 2.5 cm depth for abamectin and fluopyram compared to the non-treated control. Further, a similar number of J2 were observed at 5.0 and 7.0 cm inoculation depths for all treatments (Fig. 6B).

## Discussion

These data indicate that seed-applied abamectin and fluopyram suppressed *M. incognita* root penetration that contributed to a reduction in root galling and nematode reproduction in cotton and soybean in the greenhouse. Given that fewer *M. incognita* entered the root systems, suggest that these nematicides affected nematode motility prior to root penetration. Abamectin and fluopyram at low concentrations can inhibit *M. incognita* motility (Faske and Starr, 2006; Faske and Hurd, 2015; Heiken, 2017) and likely impede second-stage juvenile migration or orientation toward a root in coarse sandy soil. Clay particles can affect the movement of non-fumigant nematicides (Rich et al., 2004), thus suppression of J2 infection by fluopyram may be lower in soil textures with greater percentages of clay particles. The effect of soil texture on the distribution of fluopyram may account for some of the variability in root protection by seed-applied fluopyram in cotton and soybean field trials (Kandel et al., 2017; Faske et al., 2018).

There was no suppression of *M. incognita* maturity by seed-applied abamectin or fluopyram in cotton and soybean. Though fewer sausage-shaped juveniles were observed at 7 d after inoculation (DAI) for fluopyram and abamectin in soybean, the percent of maturity stages at later sample times was similar to that of the control. Soil-applied abamectin at 0.6 μg/cm^3^ for 2 d prior to being infested with 200 J2 was reported to be effective at suppressing *M. arenaria* development for 8 d in tomato (Cayrol et al., 1993). Whereas soil-applied oxamyl at 6.25 µg/cm^3^ soil applied 2 d after being infested with 200 J2 was effective at suppressing *M. incognita* development for 30 d in cucumber (Wright and Womak, 1981). While suppression of nematode development has been reported in at least one non-systemic nematicide, abamectin, no suppression was observed with it as a seed treatment.

The depth of root protection against *M. incognita* penetration was similar between nematicides and crops. Seed-applied fluopyram suppressed J2 entry up to 5.0 cm in cotton and 2.5 cm in soybean. Fluopyram as a seed treatment was reported to suppress root penetration of the soybean cyst nematode, *Heterodera glycines*, up to 2.5 cm depth (Beeman et al., 2019). Seed-applied abamectin conferred protection at the 2.5-cm depth in cotton and soybean. Similarly, seed-applied abamectin was reported to suppress *M. incognita* nematode infection at a 5 cm depth on a cotton taproot (Faske and Starr, 2007). These data indicate that suppression of *M. incognita* penetration is greatest within close proximity to the seed and decreases with increasing distance from the seed.

Fluopyram has a greater impact on plant-parasitic nematodes in the soil prior to root penetration rather than on nematode development inside the root in cotton and soybean. Thus, the movement of fluopyram from the seed coat into the nearby soil affects the depth of root protection from plant-parasitic nematode. These observations contribute the understanding of the variability in suppression of plant-parasitic nematodes by seed-applied fluopyram.
